# Description of a Retrospective Cohort of Epithelial Ovarian Cancer Patients with Brain Metastases: Evaluation of the Role of PARP Inhibitors in this Setting

**DOI:** 10.3390/jcm12072497

**Published:** 2023-03-25

**Authors:** Zena Alizzi, Patricia Roxburgh, Douglas Cartwright, Alistair McLaren, Sarah Park, Rachel Jones, Semini Greening, Emma Hudson, Clare Green, Simon Gray, Saira Khalique, Emmanouil Karteris, Marcia Hall

**Affiliations:** 1 Mount Vernon Cancer Centre, Rickmansworth Road, Northwood HA6 2RN, UK; 2 Beatson West of Scotland Cancer Centre, University of Glasgow, Glasgow G12 0YN, UK; 3 Royal Cornwall Hospitals NHS Trust, Truro TR1 3LJ, UK; 4 South West Wales Cancer Centre, Swansea SA2 8QA, UK; 5 Churchill Hospital, Oxford OX3 7LE, UK; 6 Velindre Cancer Centre, Cardiff CF14 2TL, UK; 7 University Hospital Southampton, Southampton SO16 6YD, UK; 8 Lancashire Teaching Hospitals NHS Foundation Trust, Preston PR2 9HT, UK; 9 College of Health, Medicine and Life Sciences, Brunel University London, Uxbridge UB8 3PH, UK

**Keywords:** epithelial ovarian cancer, brain metastasis, PARP inhibitors

## Abstract

Background: The incidence of brain metastases (BM) in patients with epithelial ovarian cancer (EOC) is low: 0.3–11%. The onset of BM has been regarded as a late event with limited treatment options and poor prognosis. This retrospective case series aims to explore the current management strategies with particular emphasis on the use of PARP inhibitors and outcomes, as well as identification of other prognostic indicators. Methods: A total of 39 ovarian cancer patients with brain metastases were identified from eight cancer centres in the UK. Clinical characteristics, details of management, and survival data were collected. Results: A total of 14/39 had BM as their first site of relapse. The majority (29 patients) received systemic treatments in addition to local radiotherapy (RT)/surgery. Nineteen patients had *BRCA* mutations (one somatic), one had a *RAD51C* mutation, and eighteen were BRCA wild type; one was unknown. A total of 14/39 patients received maintenance PARP inhibitors. As is well known, patients who received PARPi had consistently better outcomes. This was no different for those who received PARPi as part of the management of their BM. Platinum sensitivity and receiving more than one modality of therapy (e.g., radiation +/− chemotherapy and PARPi) for BM were also good prognostic indicators. Median PFS/OS for those treated with chemotherapy and either RT or surgery, then PARP inhibitor maintenance, have not been reached after a median of 33 months follow up. Conclusions: As with abdominal relapse, maintenance treatment with PARP inhibitors also has a valuable role in managing BMs in EOC patients.

## 1. Introduction

Epithelial ovarian cancer (EOC) is the fifth most common cancer among women in the UK (2011), accounting for 6% of female cancer deaths [1]. EOC typically presents at an advanced stage, with 70% patients being diagnosed at Stage III/IV. Unlike lung and breast carcinomas, where brain metastases (BM) are common [2,3], the reported incidence of BMs in EOC patients ranges from 0.3–11% [4,5]. However, better clinician awareness and improvements in systemic therapies and imaging techniques have led to an increasing incidence [6]. According to published data, the time from diagnosis to the development of BMs in EOC patients ranges from 11 to 46 months, and median survival is approximately 8–12 months from diagnosis of BM [4,7,8]. BM have been associated with a poor prognosis, exacerbated by concurrent systemic disease recurrence [9]. Poor prognostic indicators include more than one brain lesion, platinum resistance, and wild-type germline *BRCA* (*gBRCAwt*) status [10]. To date, there is no accepted optimal treatment strategy. Given their rarity, the only available evidence to support management decisions in this group is likely to be retrospective.

Current treatment approaches include radiotherapy (RT), surgery, and chemotherapy; more recently, stereotactic radiosurgery (SRS) is often employed. SRS is widely used to treat small BM/oligometastatic disease. The outcomes are more favourable in terms of toxicity with very little neurological compromise [11]. Data also supports prolonged overall survival (OS) following SRS, but the highly selected patient cohort (single site of disease, good disease control, and good performance status) may influence these outcomes [12]. Whole-brain radiotherapy (WBRT) is frequently used for patients with multiple BM; however, it is associated with significant toxicities and negatively impacts quality of life [13]. The literature suggests that median survival ranges between 3 and 6 months after WBRT.

Surgery requires patients to have a good performance status, usually a single site of metastasis, and good tumour accessibility [13]; hence, outcomes are also skewed by selection bias. Median OS following surgery alone for BM was 9 months in a series of 583 cancer patients [13]. Many reports have concluded that the addition of radiotherapy (typically WBRT) to surgery improves OS for patients with EOC, from approximately 6 months with either modality alone to ~18–22 months [9,13]. However, extra-cranial disease and/or multiple BM at relapse precludes surgery for many EOC patients and highlights the need to explore different treatment modalities to improve outcomes.

Chemotherapy is generally the treatment of choice for extra-cranial disease in patients with EOC; yet, there are very few reports where chemotherapy is used alone in EOC patients with BMs. However, chemotherapy is often administered as ‘adjuvant’ following ‘definitive’ RT/surgery. For example, in an Italian case series of EOC patients with BM, 63.3% received chemotherapy but very few (4.6%, 8/174) were treated with chemotherapy alone without additional surgery/RT [8]. Poly-ADP ribose polymerase inhibitors (PARPi), have been shown to be beneficial in patients with EOC, especially those with homologous repair deficiency (HRD), but their use has not been investigated in the context of BM. At the time of this study, in the UK, PARPi were only available for patients with platinum-sensitive relapsed ovarian cancer, outside of clinical trials. This case series aims to explore the different prognostic indicators and current treatments used in EOC patients with BM, and report patient outcome. In particular, the use of PARPi for brain metastases in this context is evaluated.

## 2. Materials and Methods

### 2.1. Patient Selection

The advent of widespread PARPi use in patients with EOC prompted discussions about their value in those with BM. In December 2019, ovarian cancer teams, in UK cancer centres, were invited to contribute to this case series, reporting management and outcomes for ovarian cancer patients with BMs. Caldicott Guardians (ethical resource) at each NHS site were consulted and informed consent for this retrospective chart review was waived. Details of 39 non-consecutive patients, all diagnosed and treated in the last 15 years, were submitted for inclusion. Brain metastases were diagnosed in these patients between April 2014 and January 2021. Eight UK gynaecological cancer centres contributed to the series. The Beatson West of Scotland Cancer Centre submitted details of the largest number of patients (n = 17), with 12 from Mount Vernon Cancer Centre (MVCC).

### 2.2. Data Collection and Outcome

Baseline data was collected from patient records made at standard follow-up visits, generally ~3 monthly and more often during treatment periods. Data collected consisted of history of diagnosis and treatments for EOC, time of onset of BM, number of BM, presence/absence of extra-cranial disease, stage, histology, *BRCA* status (germline *BRCA* mutation—*gBRCAm,* somatic *BRCA* mutation—*sBRCAm*), and primary and relapse treatments. The interval between last treatment for EOC and BM was determined. Patients relapsing more than 6 months from last platinum-based therapy were classed as platinum sensitive and those who relapsed less than 6 months were classed as platinum resistant. A strict template for data collection was completed by all sites.

### 2.3. Statistical Analysis

Statistical analysis was carried out using GraphPad Prism software to calculate univariate and multivariable analyses. The Kaplan–Meier method was used to calculate survival curves. *p* value < 0.05 was used for statistical significance in all analyses. In particular, given the well-known disparities in outcomes in specific groups of EOC patients without BM, the differences between those with and without *BRCA* mutations, with and without extra-cranial disease, and the platinum-free intervals were sought. Progression free survival (PFS) was calculated from the time of first treatment for brain metastases to further disease progression. Overall survival (OS) was calculated from the BM diagnosis until death/last follow up.

## 3. Results

### 3.1. Baseline Clinical Characteristics

**Figure 1:**
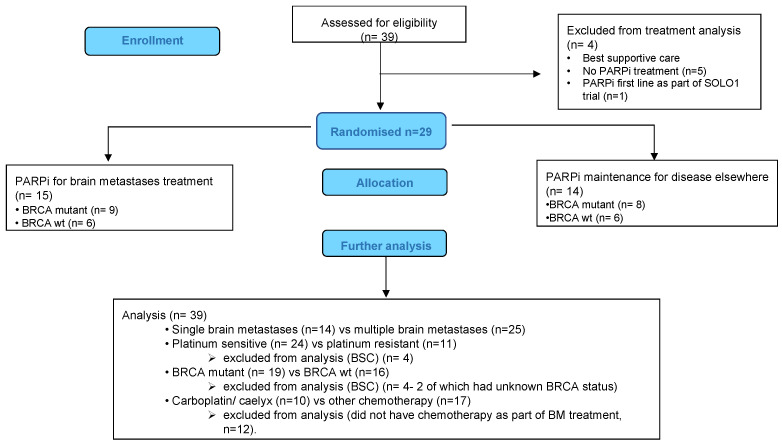
CONSORT diagram to describe patient groups.

A total of 39 patients from eight UK cancer centres were submitted. Four patients had best supportive care and were excluded from treatment analyses (Figure 1). Clinical characteristics are summarised in Table 1. The median age at diagnosis of EOC was 65 years (44–84 years). All patients were diagnosed with high-grade serous EOC and the majority (35/39) presented with advanced stage (FIGO IIIC/IV) when diagnosed. Clinical characteristics are summarised in Table 1.

### 3.2. Treatment Modalities and Survival

**Figure 2:**
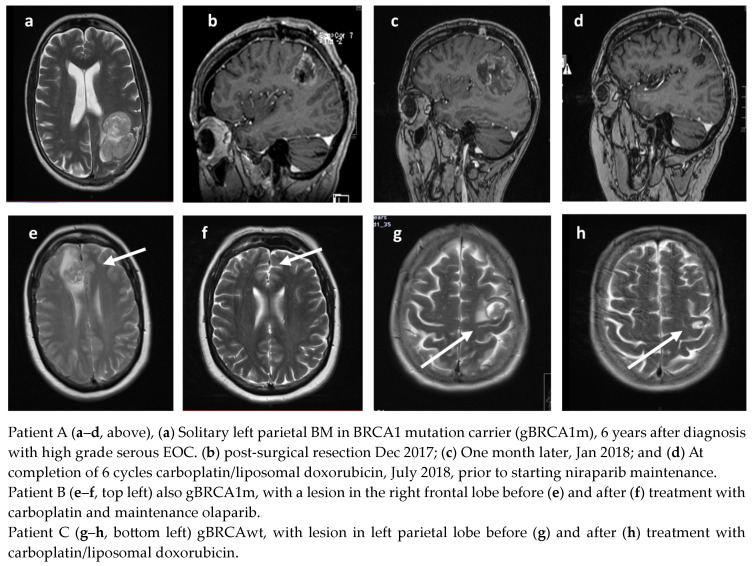
Radiological responses of three patients with BM on PARPi therapy (arrows denote sites of brain metastases).

A total of 35 patients received treatment for BM. The median time to BM from the end of first-line treatment for *BRCAm* patients was 21.5 months (range 8–42), and 23 months (range 16–27) for *BRCAwt* patients. BM as the only site of first relapse occurred in 14 patients (*BRCAm* n = 7 and *BRCAwt* n = 7). Thirteen of these patients had an initial remission of greater than 1 year (13–52 m) prior to their relapse with BM. Systemic treatments were not planned for five of these fourteen BM patients, although one patient responded well to chemotherapy having progressed within six weeks of surgery (Figure 1). A total of 7/14 patients (three *BRCAm*) had further relapse(s) following treatment, with a median PFS of 13.5 months (2–25 months). At the time of analysis, 6/14 (four *BRCAm*) remain in remission, with a median PFS of 37 months (10–64 months), following treatment for BM. Four remain on PARPi. Examples of patients are demonstrated in Figure 2a–h.

Patient A, with *gBRCA1m*, was diagnosed with a solitary BM 42 m after initial diagnosis (Figure 2a). The left parietal lobe mass was surgically resected (Figure 2b), but she presented one month later with deteriorating neurology; her performance status had declined to three. Repeat MRI demonstrated early recurrence in the surgical bed and a new right parietal metastasis (Figure 2c). She was treated with six cycles of carboplatin/liposomal doxorubicin followed by maintenance niraparib; she remains in complete remission (CR) over 36 months later and is still taking niraparib (Figure 2d). Patient B, also *gBRCA1m*, was treated with carboplatin and maintenance olaparib (Figure 2e,f). Patient C, *gBRCAwt,* received caboplatin/liposomal doxorubicin and maintenance niraparib (Figure 2g,h).

Table 2 demonstrates the treatment modalities delivered for BM in the 35 patients. Of the 14 patients with BM only at first relapse, 1 patient, *BRCAwt*, received six cycles of carboplatin alone and has remained in CR for 60 months. Three further patients were treated with chemotherapy and PARPi. One patient had combination chemotherapy alone, and the remaining nine were treated with combinations of surgery then radiotherapy (n = 2), surgery and chemotherapy (n = 1), surgery, chemotherapy and PARPi (n = 4), and one each with surgery/radiotherapy/chemotherapy and radiotherapy/chemotherapy/PARPi. No patient received all four modalities (surgery/radiotherapy/chemotherapy and PARPi) but the patient who had surgery/radiotherapy and chemotherapy for her first relapse BM went on to receive single agent carboplatin and maintenance PARPi treatment at a subsequent relapse with brain and peritoneal disease.

Of 21 patients with recurrent extra-cranial disease, prior to developing BM, 9 patients were *BRCAm* carriers, 1 patient had a pathogenic *RAD51C* mutation, and 10 patients were *BRCAwt*. In one case, the *BRCA* mutation status was unknown. Four patients presented with extra-cranial disease and BM together at first relapse; three were treated with carboplatin-based chemotherapy, two of whom had PARPi following complete and partial responses. One patient did not respond to chemotherapy and died shortly after the fifth cycle. The remaining patient had surgery and radiotherapy and remains in remission at data cut off.

Eight of these twenty-one patients developed BM at second relapse, nine at third relapse, three at fourth relapse, and one patient at eighth relapse (all had pre-existing extra-cranial disease). Three patients were treated with surgery/radiotherapy and two received WBRT; seven had chemotherapy, three had chemotherapy/radiotherapy, and the remaining patients were managed with letrozole (n = 1) and BSC (n = 4). The patient who relapsed eight times was ultimately found to have a *RAD51C* mutation at the time of her BM; she has been on rucaparib since this time, although has had repeat stereotactic radiosurgery (SRS) for a solitary recurrent BM (30 m).

A total of 6/9 (66%) patients with *BRCAm* had received PARPi treatment prior to the development of their BM—three olaparib, one niraparib, one rucaparib, and one patient had veliparib and olaparib. By contrast, 5/12 (42%) *BRCAwt* patients had had prior exposure to PARPi.

### 3.3. Effects of PARP Inhibitors on Overall Survival of EOC Patients with BM

**Figure 3:**
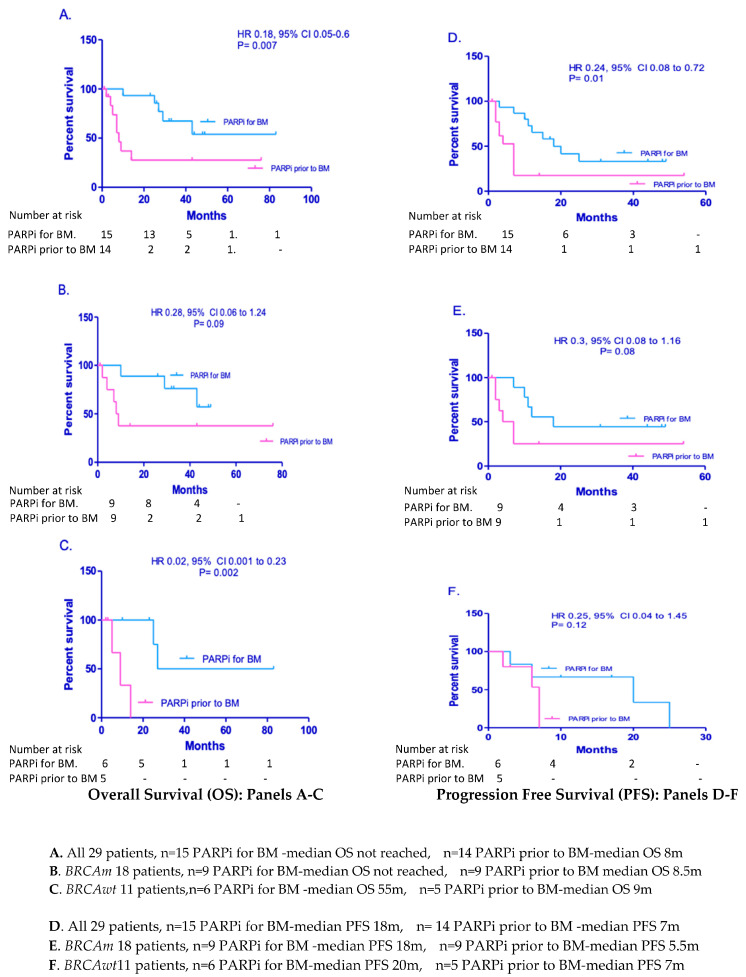
OS (**A**–**C**) and PFS (**D**–**F**) of EOC patients with BM according to PARPi for BM vs. PARPi prior to BM.

A total of 29/35 patients received PARPi treatment as part of managing their OC. Fifteen EOC patients (nine *BRCAm*, six *BRCAwt*) received PARPi as part of managing of BM: seven were treated with niraparib, eight with olaparib, and two patients received rucaparib. Of these 15, 13 had BM as their first (platinum sensitive) relapse, median PFS was 20 m, and median OS had not yet been reached (Figure 3A,D). The remaining 14 patients (six *BRCAwt*, eight *BRCAm*) received PARPi but as maintenance following chemotherapy for disease elsewhere. In 13, this was prior to the development of BM. One *BRCA1m* patient in this group had BM treated with surgery/RT and four years later received niraparib for abdominal disease relapse. Following treatment for BM in these 14 patients, the median PFS was 7 m (Figure 3A,D). The superior survival for those who received PARPi for BM is most likely attributable to the fact that 13 of 15 patients in this group had BM as their first platinum-sensitive relapse compared with the “PARPi prior to BM” group where outcomes had been measured from treatment for BM which were second or subsequent relapses.

Analysing the *BRCAm* and *BRCAwt* groups individually, access to PARPi therapy for BM management significantly improved OS for those with *BRCAm* (*p* = 0.03, HR 0.17 95% CI 0.04–0.8) (Figure 3B,C). There was a similar trend for six *BRCAwt* patients, who were managed with PARPi as part of their treatment for BM, median OS of 55 m, compared with those *BRCAwt* patients who had had PARPi prior to development of BM: median OS 7 m, but this did not reach significance (*p* = 0.1, HR 0.29 95% CI 0.07–1.25). Although there were longer median PFS in both *BRCAm* and *BRCAwt* patients where PARPi were used for BM compared with the population who received PARPi prior to BM, this did not reach significance. These were 18 m vs. 7 m (*p* = 0.12 HR 0.38 95% CI 0.11–1.3) for the *BRCAm* population and 25 m vs. 6 m (*p* = 0.23 HR 0.47 95% CI 0.14–1.6) for the *BRCAwt* group (Figure 3E,F). 

### 3.4. Other Possible Prognostic Factors Such as Platinum Sensitivity, BRCA Mutation Status, and Number of Treatment Modalities Undertaken

**Figure 4:**
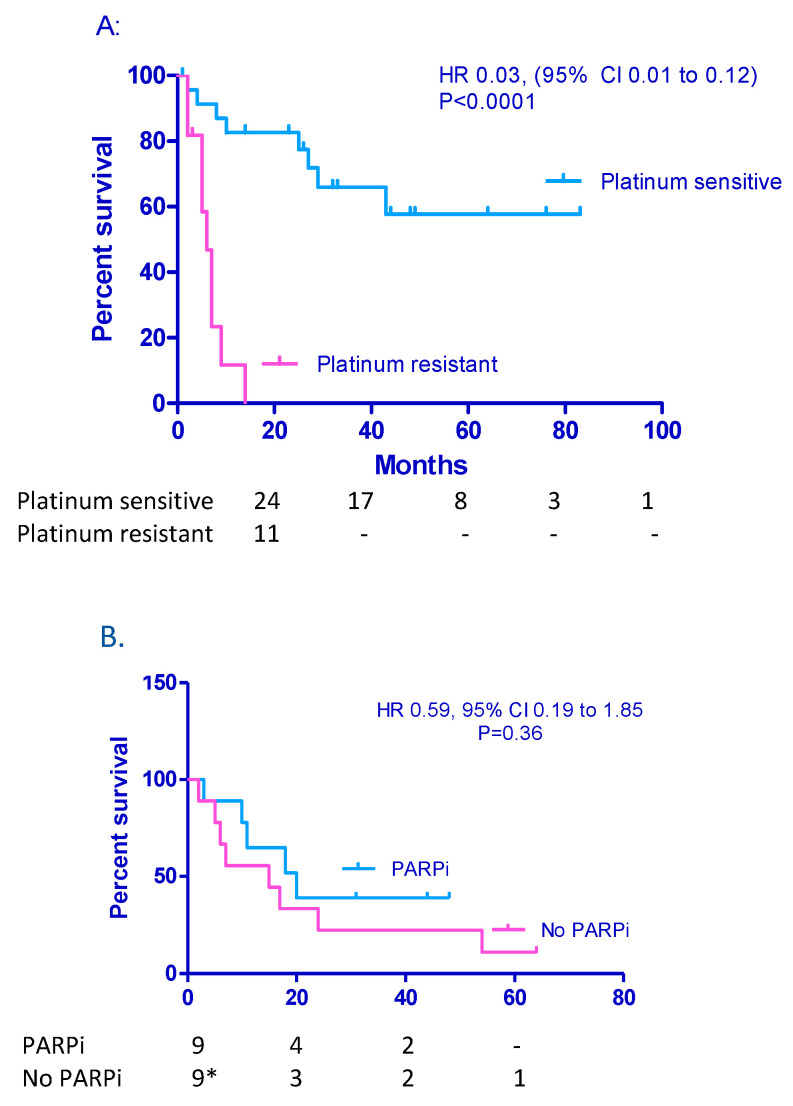

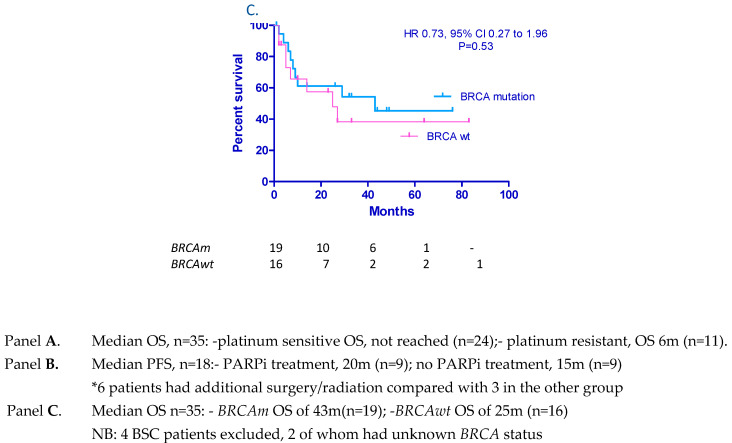
Median OS according to platinum sensitivity (**A**), PARPi vs. no PARPi maintenance treatment in platinum sensitive relapse cohort (**B**), and *BRCA* status (**C**).

There was no significant difference in OS between those who had single or multiple BM (43 m vs. 14 m, respectively, *p* = 0.96 HR 1.6, 95% CI 0.64–3.89). However, platinum sensitivity was highly prognostic for survival. Median OS was not reached in those patients who had had greater than a 6-month interval since their last platinum therapy compared to 6 m in the platinum resistant group (Figure 4A). Of 18 patients with platinum sensitive relapse for their BM (17 first relapse, 1 patient second relapse): 9 (6 BM alone, 3 with additional peritoneal/nodal relapse) received maintenance PARPi and 9 (no-one had disease outside BM) did not receive maintenance PARPi. There was no significant difference in PFS (Figure 4B); however, of those who did not receive maintenance PARPi, five patients received surgery, three in combination with radiation and two in combination with CT, and one patient received radiation with CT. In contrast, of those who received maintenance PARPi, only three patients had additional surgery (no radiation). All these patients received CT prior to PARPi.

Of the 37 patients where BRCA status was known, 18 patients were *BRCA* mutant carriers: 14 *BRCA1m* and 5 *BRCA2m*. Median OS was 43 m for patients with *BRCAm* vs. 25 m for those who were *BRCAwt* (*p* = 0.53) (Figure 4C).

Radiation and surgery are frequently considered as treatments of first choice when patients present with BM. The introduction of chemotherapy and PARPi have broadened this choice, although they are not often considered initially by clinicians without a particular interest in gynaecological malignancies. We compared the outcomes of patients, according to how many modalities of therapy they received (Table 3). A total of 14 of 35 patients were managed with only one modality of treatment: chemotherapy in 10, radiation only in 13, and 1 patient (*RAD51C*m) responded to single-agent rucaparib for more than 24 months, at her eighth relapse. These 14 patients were used as our reference control group. Seven patients with BM were managed with a second non-PARPi modality and, although the median OS was better, this did not reach statistical significance. However, for the patients whose treatments included PARPi therapy, the median OS was statistically better whether they had additional chemotherapy alone (i.e., doublet therapy, n = 8) or triplet therapy (i.e., additional radiation/surgery, n = 6).

Caution is required considering these results as the majority of those receiving PARPi as part of the management for BM (chemotherapy + PARPi, chemotherapy + PARPi + other) were at first platinum-sensitive relapse compared with only 5/21 in the ‘no PARPi’ groups (single therapeutic intervention and doublet, no PARPi). Of the remaining 16/21 patients, 3 never received PARPi at all and had BM at third relapse, 9 had received PARPi for first abdominal relapse and no PARPi for BM developed at later lines (median third line), and 3 had received PARPi at 2/3 relapse with BM developing at fourth relapse. One patient had received olaparib as part of SOLO1 and her second relapse included BM.

## 4. Discussion

Clinical experience suggests that EOC patients carrying *BRCAm* have a higher incidence of BM. Ratner et al. report that 3% of the patients with *BRCAm* developed BM compared with 0.6% of those who were *BRCAwt* from a large study of >4500 EOC patients [14]. A more recent retrospective study from Israel, reviewing 1035 patients since 2002, suggests that there are more EOC patients overall developing BM with a prevalence of 5.1% in those with *BRCAm* and 2.1% in those who are *BRCAwt* [15]. This is likely to be due to the availability of more and improved treatments for patients with EOC including maintenance PARPi. However, there is limited published evidence on the efficacy of treatment options for EOC patients with BM. A multimodal approach is considered optimal, where feasible, on the basis of a study of 174 patients with a spectrum of solid tumours where median OS was ~22 m with triplet modality therapy compared with 5 m from a single treatment type [13]. Nevertheless, there is always an element of patient selection in such survival data; those with poorer performance status are generally not offered more than one modality. Historical data focus on localized treatment modalities such as RT and surgery, and there continues to be doubt about the efficacy of systemic treatments, particularly regarding their ability to cross the blood–brain barrier (BBB) [13].

Published evidence suggests that patients with multiple BM have poorer outcomes, but possibly this reflects the historic use of localized treatments such as radiation/surgery without systemic therapy [10]. In this series, patients with multiple brain lesions did not have significantly shorter OS in comparison to those with single-site intracranial disease (Figure S1). This is likely to be due to the large number of patients, from the two busiest cancer centres, having systemic treatment such as chemotherapy/PARPi with only six patients receiving localized surgery/radiation alone for BM (two with single-site BM). There are many case reports of the utility of chemotherapy in EOC patients with BM, but this is the largest series presented since the introduction of PARPi therapy into routine clinical practice. However, there is little data on the choice of chemotherapy agents for EOC patients with BM [8]. Of the 29 patients who received chemotherapy for BM in this series, 12 (41%) received carboplatin/liposomal doxorubicin. Doxorubicin has been shown to be one of the most effective agents against glioma cells in vitro [16] but, because of its poor BBB penetration, is not used in this context. The development of the liposomal preparation of doxorubicin, with evidence of significant improvements in BBB penetration in animal models [17], prompted Fabel et al. to explore the value in patients with recurrent high-grade gliomas [18]. Disease stabilization was observed in 54% in a small series of 13 patients. In this series of EOC patients, the 12 patients who received liposomal doxorubicin for BM had a longer PFS (16 m vs. 7 m) and OS (43 m vs. 27 m) than patients treated with alternate chemotherapy agents; seven of these patients received this at first relapse and the remainder at later lines of relapse. (Figure S2).

There are substantial barriers to ascertaining the value of maintenance PARPi in EOC patients with BM. It is well known that PARPi improve outcomes for patients with *BRCAm*, but the evidence for PARPi in controlling BM is less clear. Here, the comparison of two small groups of patients, all of whom have received PARPi at some point, half of them specifically as part of their BM treatment, suggests that PARPi maintenance does add similar benefits and improves the median OS in a BM group in comparison with the historically reported 2–7 month median OS following chemotherapy alone [19]. This suggests that PARPi can cross the BBB in vivo, as demonstrated in in vitro models [20,21]. Prior case reports of PARPi in EOC patients with BM have mostly been limited to those with *BRCAm*, with the range of PFS reported as 9–22 m [22]. Our data, with a median PFS of 18 m, substantiates this. Additionally, we demonstrate an advantage for a number of *BRCAwt* patients, in correlation with Zhang et al. [23] who describe a PFS of 29 m in their case report of a *BRCAwt* patient treated with niraparib. Unfortunately, as with the Zhang report, HRD tissue testing was not available for any of our patients, and it is possible that the benefits are limited to those with HRD.

Median OS for patients who were platinum sensitive at the time of treatment for BM had not been reached at the time of data cut off and was statistically different from the 6 m median OS seen in those who were platinum resistant (Figure 3A). These results support the findings of a German multicentre retrospective series where they also demonstrate that platinum sensitivity and *BRCAm* status remain good prognostic indicators in this setting [24].

The *BRCAm* patients here presented with BM an average of 8 months earlier than *BRCAwt* patients, suggesting that this development is not related to the better outcomes of patients with DNA repair deficits. The limited literature with regard to survival amongst *BRCAm* patients with brain metastases is conflicting. Ratner et al. report a median OS of 7 m (3.5–17 m) from diagnosis of BM in 46 patients, with no significant difference in relation to *BRCAm* status [25]. Limon et al. report a median OS of 20.6 vs. 12.3 months (*p* = 0.44) following diagnosis of BM in patients with and without *BRCAm,* respectively, although, again, not significant [15]. Both are shorter than the median OS described in this analysis (Figure 3): 43 m in *BRCAm* vs. 25 m in *BRCAwt* patients (*p* = 0.53), perhaps attributable to PARPi use in both groups. Balendran et al. found that by using next generation sequencing of BM in OC, the most common altered genes were BRCA 1/2, TP53, and ATM. They demonstrated that in all cases there was at least one mutation present in a DNA repair gene, confirming this as a risk factor for BM development [26] and supporting the use of PARPi in the *BRCAwt* population with BM. There is similar evidence that HRD in patients with breast cancer increases their risk of BM [27]. If HRD is a risk factor for BM, this may explain the similarities in OS between the *BRCAwt* and *BRCAm* populations here, as some *BRCAwt* patients may have had mutations in alternative HRD genes. A less plausible hypothesis is that differing biology causes BM earlier in *BRCAm* patients, limiting OS to equal that of a *BRCAwt* population.

The usual limitations (e.g., recollection bias) besetting retrospective studies apply here. Other weaknesses include potential confounding variables and effect modifiers such as the lack of inclusion of brain imaging when restaging patients with relapsed EOC. This possibly reduces the accuracy of the time of diagnosis of BM in relation to any individual’s EOC journey. Other drawbacks include a lack of data on performance status and quality of life. Only one patient had somatic testing in this series, following 6–7 platinum-sensitive relapses. She had a pathogenic *RAD51C* mutation. However, there was no access to routine HRD or somatic *BRCA* testing during the time in which this series of patients were treated. HRD/somatic *BRCA* mutations may explain the better outcomes that we report here for those who are *BRCAwt*. It will be important to evaluate the value of chemotherapy and PARPi again in patients with full HRD/somatic *BRCAm* information. Equally valuable will be to explore the outcomes of the *BRCAm* and HRD population who relapse with BM in the future but who are ineligible to receive further PARPi because they have already received them as maintenance first-line treatment.

## 5. Conclusions

In conclusion, this series demonstrates good disease control from multimodality treatment approaches in EOC patients with BM, irrespective of *BRCA* status. Patients selected for combination, systemic, and localized treatment, stereotactic RT/surgery if the BM are suitably accessible, followed by maintenance PARPi fared best. If patients are not fit enough, or the BM are not accessible for localized treatments, platinum-based chemotherapy with PARPi maintenance can be effective.

## Figures and Tables

**Table 1:** Baseline clinical characteristics of 39 patients with EOC and BM.

*BRCA* Status	Number of Patients
Unknown	2
gBRCA 1 mutant	13
gBRCA 2 mutant	4
sBRCA mutant	2 (one of each)
WT	18 (1 RAD51Cm)
Stage (FIGO)	
II	4
III	17
IV	18
Type of initial surgery	
Primary debulking surgery	19
Interval debulking surgery	15
No surgery	5
Number brain metastases	
1	12
2	2
Multiple (≥3)	25
Extra-cranial disease with first BMs	
No	14 *
Yes	25 ^†^

* (7/14 *BRCA*m), ^†^ (11/25 *BRCA*m, 1 *RAD51C*m).

**Table 2:** Details of relapse with BM and treatments delivered.

Line of Relapse with First BM		Number of Patients (n = 39)
	1st relapse	18
	2nd relapse	8
	3rd relapse	9
	≥4th relapse	4
Platinum sensitivity at first BM		
	Sensitive (>6 months)	24 (15 *BRCAm*)
	Resistant (<6 months)	15 (4 *BRCAm*, 1*RAD51Cm*)
PARP inhibitor treatment		
	None	9
	Before diagnosis BM	14 ^#^
	For BM management	15
Treatment of first BM any relapse n = 35 *		
	Chemotherapy alone	10 (1 first relapse BM only)
	Radiotherapy alone	3
	PARP inhibitor only	1 ^†^
	Radiation/chemotherapy	2
	Surgery/chemotherapy	1
	Surgery/radiation	4
	Chemotherapy/PARPi	8
	Radiation/chemo/PARPi	1
	Surgery/chemo/PARPi	4
	Surgery/radiation/chemo	1

^#^ One had olaparib first line as part of trial and one patient had maintenance PARPi for extra-cranial disease whilst previously treated BM were still in CR, * four best supportive care, ^†^ eighth relapse found to have *RAD51C*m.

**Table 3:** Median OS in relation to number of treatment modalities received for BM. NB: 13/14 those receiving PARPi for BM were first platinum-sensitive relapse, compared with 5/21 who did not receive PARPi for BM at first relapse.

Treatment	Number (n = 35 ^†^)	Median OS (Months)	*p*
Single therapeutic intervention *	14	5.5	-
Doublet (no PARPi)	7	23	NS
Chemotherapy + PARPi	8	26.5	0.05
Chemotherapy + PARPi + other	6	44	0.03

* Single therapeutic intervention: either radiotherapy or surgery or chemotherapy. ^†^ Excludes four patients who were managed with best supportive care.

## Data Availability

Data will be available upon reasonable request.

## Supplementary Materials

The following supporting information can be downloaded at: https://www.mdpi.com/article/10.3390/jcm12072497/s1, Figure S1. Median OS according to single vs. multiple BM; Figure S2. OS (left) median 43 m vs. 27 m and PFS (right) 16 m vs. 7 m liposomal doxorubicin/carboplatin vs. other chemotherapy respectively for BM.
