# Supplementary material for: Description of a Retrospective Cohort of Epithelial Ovarian Cancer Patients with Brain Metastases: Evaluation of the Role of PARP Inhibitors in this Setting
Source: J Clin Med. 2023 Mar 25;12(7):2497. doi: 10.3390/jcm12072497 (PMC10095324; doi:10.3390/jcm12072497)

**Supplementary Figure S1**: Median OS according to single vs multiple BM

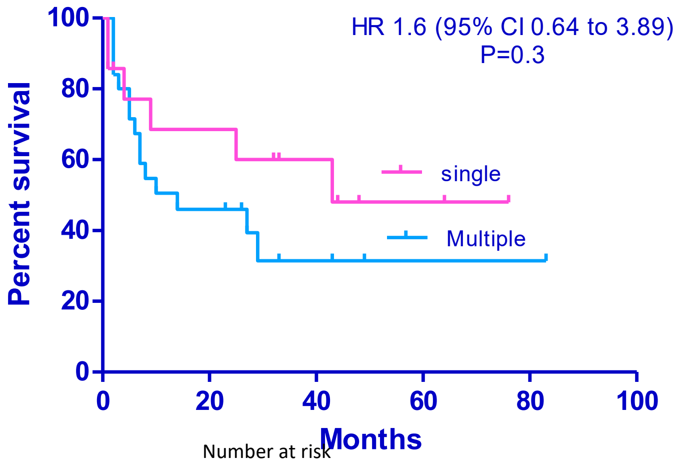


Number at risk

| | | | | | |
|---|---|---|---|---|---|
| Single BM | 14 | 8 | 5 | 2 | - |
| Multiple BM | 25 | 9 | 2 | 1 | 1 |

**Supplementary Figure S2**: OS (left) median 43m vs 27m and PFS (right) 16m vs 7m liposomal doxorubicin/carboplatin versus other chemotherapy respectively for BM

PFS:

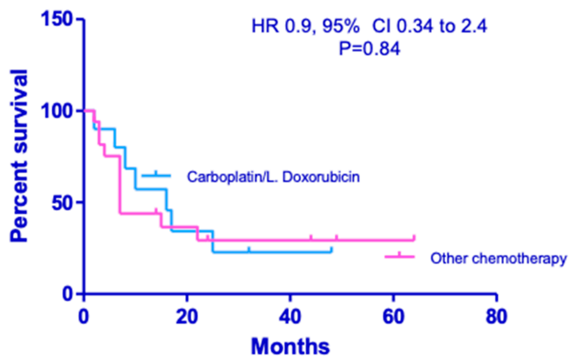

Supplement: Supplementary file 1 [file jcm-12-02497-s001.zip › jcm-2111954-SI.pdf]
